# Supplementary material for: Regulation of KIF2A by Antitumor miR-451a Inhibits Cancer Cell Aggressiveness Features in Lung Squamous Cell Carcinoma
Source: Cancers (Basel). 2019 Feb 22;11(2):258. doi: 10.3390/cancers11020258 (PMC6406917; doi:10.3390/cancers11020258)
Supplement: Supplementary file 1 [file cancers-11-00258-s001.pdf]

cancers

MDPI

Supplementary Materials: Regulation of *KIF2A* by Antitumor *miR-451a* Inhibits Cancer Cell Aggressiveness Features in Lung Squamous Cell Carcinoma

Akifumi Uchida, Naohiko Seki, Keiko Mizuno, Yasutaka Yamada, Shunsuke Misono, Hiroki Sanada, Naoko Kikkawa, Tomohiro Kumamoto, Takayuki Suetsugu and Hiromasa Inoue

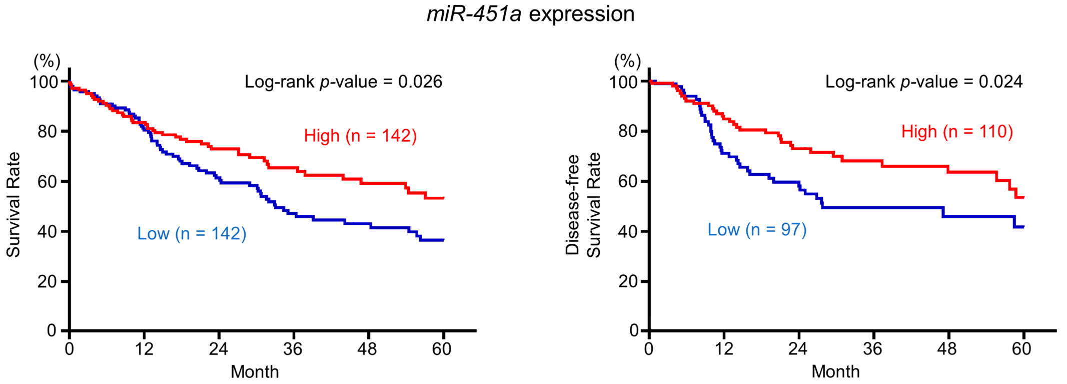

Figure S1. Kaplan-Meier analysis of *miR-451a* expression among LUSQ patients with adjusting clinical stage and age distribution. Kaplan-Meier curve of 5-year overall survival and 5-year disease-free survival according to *miR-451a* expression among patients with LUSQ in TCGA database (p = 0.026 and p = 0.024, respectively). Clinical stage and patient's age were adjusted and analyzed. Patients were divided into high (red) and low (blue) expression groups.

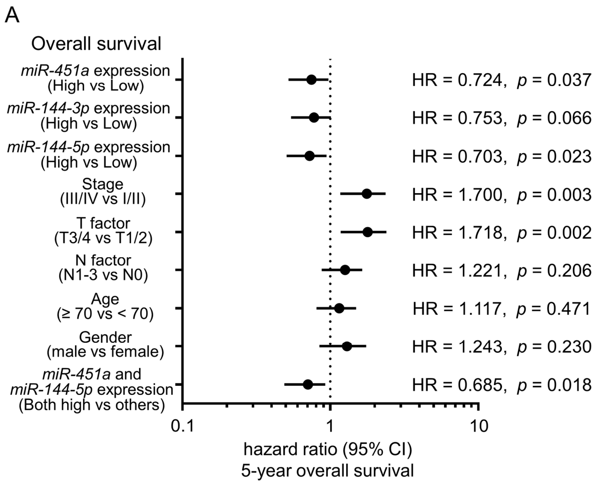

B

Overall survival

Multivariate cox proportional hazards regression analysis

	hazard ratio	95% CI	*p*-value
miR-451a	–	–	0.708
miR-144-5p	0.697	0.514 – 0.946	0.021
Stage	–	–	0.143
T factor	1.760	1.243 – 2.492	0.001

	hazard ratio	95% CI	*p*-value
miR-451a and *miR-144-5p*	0.687	0.501 – 0.942	0.020
Stage	–	–	0.151
T factor	1.737	1.226 – 2.460	0.002

Figure S2. Clinical significance of expression of *miR-451a*, *miR-144-3p* and *miR-144-5p* in the patients with LUSQ. (A) Forest plot of univariate Cox proportional hazards regression analysis of 5-year overall survival. (B) Multivariate Cox proportional hazards regression analysis of 5-year overall survival using TCGA database. High expression of 2 miRNAs (*miR-451a* and *miR-144-5p*) was an independent predictive factor for survival of the patients. For this analysis, patients were divided in half by expression levels of miRNA among TCGA database and were merged by patients ID.

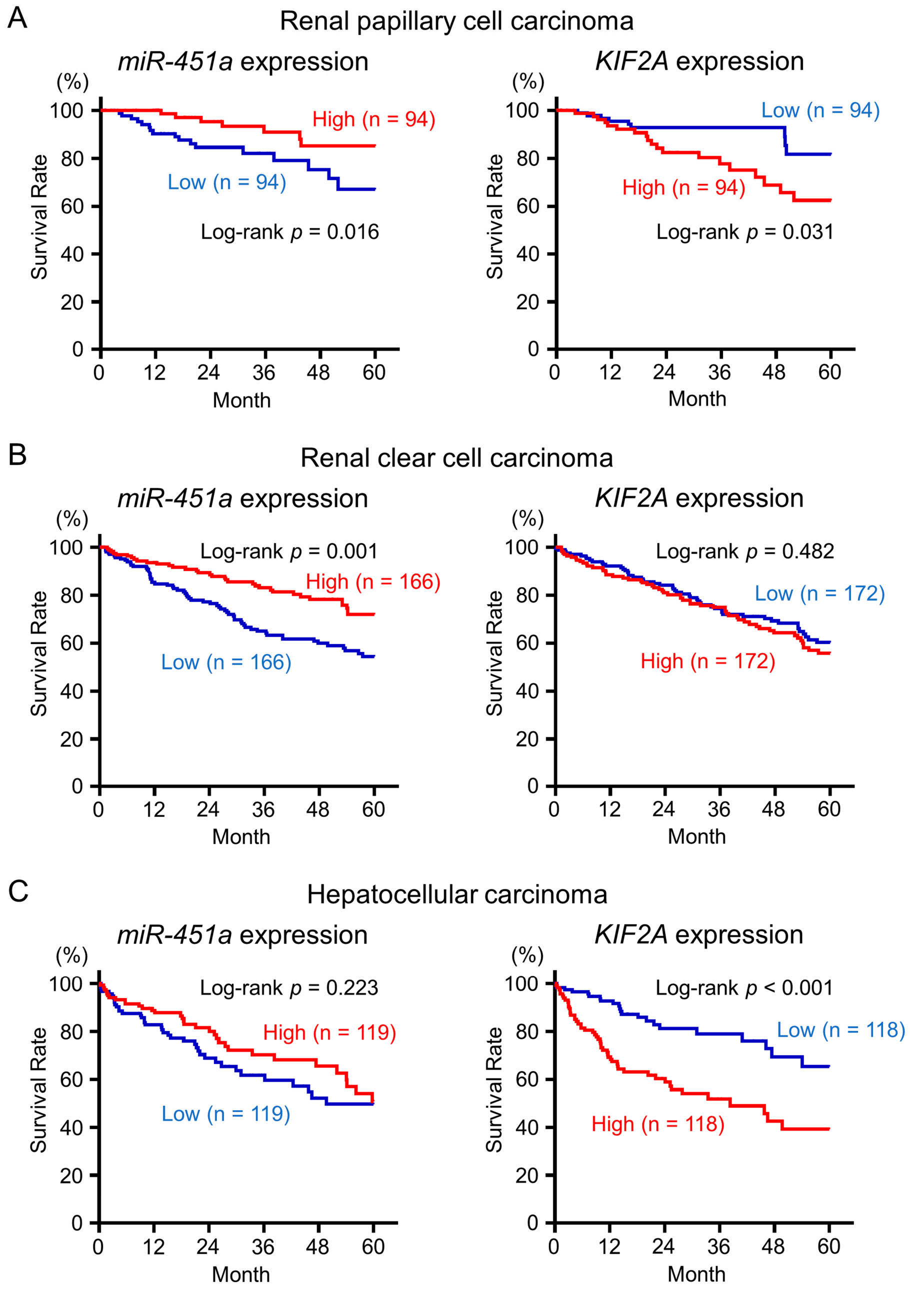

Figure S3. Kaplan-Meier analyses of *miR-451a* and *KIF2A* expression among other types of cancers. Kaplan-Meier curve of 5-year overall survival according to *miR-451a* and *KIF2A* expression among patients with renal papillary cell carcinoma (A), renal clear cell carcinoma (B), and hepatocellular carcinoma (C). Patients were divided into high (red) and low (blue) expression groups.

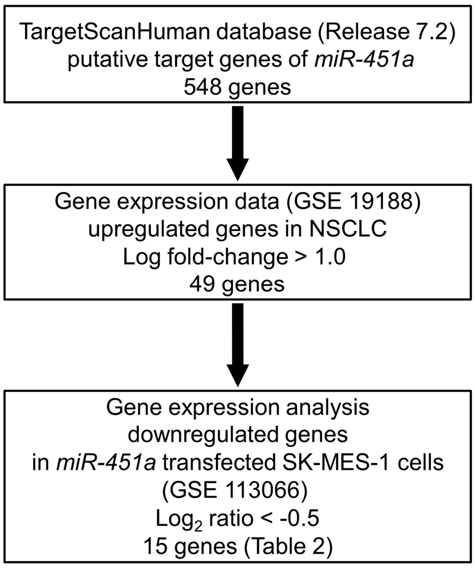

Figure S4. Flowchart illustration of the strategy for identification of target genes of *miR-451a*. By combination of *in silico* database exploration and gene expression analysis using *miR-451a*-transfected SK-MES-1 cells, we identified 15 candidate target genes.

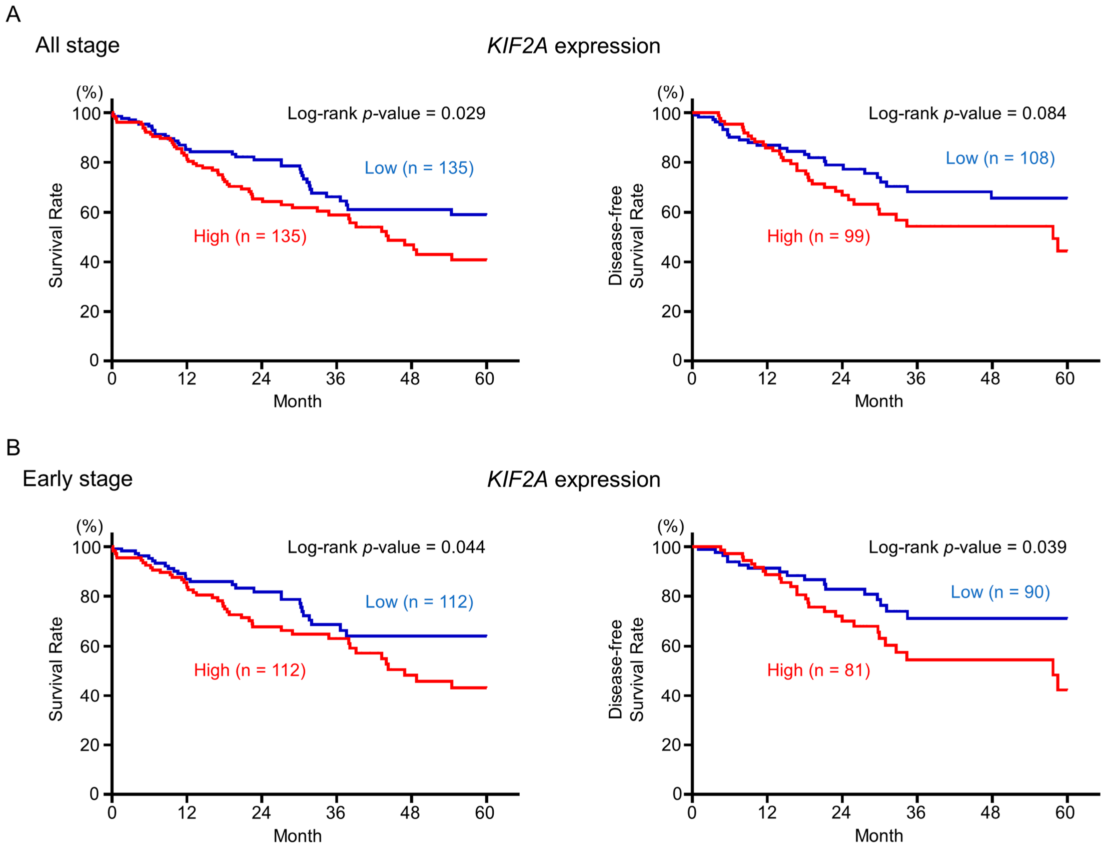

Figure S5. Kaplan-Meier analyses of *KIF2A* expression among LUSQ patients with adjusting clinical stage and age distribution. (A) Kaplan-Meier curve of 5-year overall survival and 5-year disease-free survival according to *miR-451a* expression among patients with LUSQ in TCGA database (p = 0.029 and p = 0.084, respectively). Clinical stage and patient's age were adjusted and analyzed. Patients were divided into high (red) and low (blue) expression groups. (B) Kaplan-Meier curve analyses using early stage patients of LUSQ (stage I and II). Patients were divided into high (red) and low (blue) expression groups.

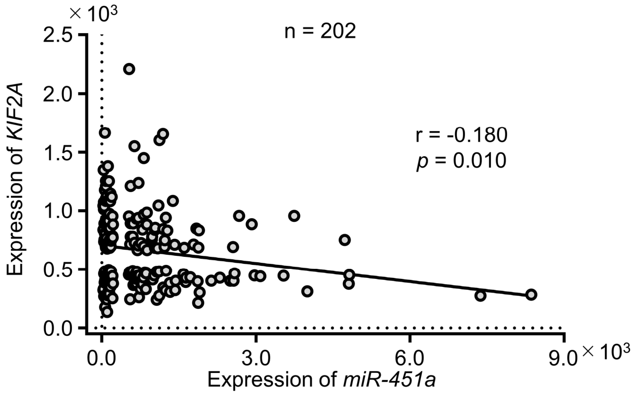

Figure S6. Correlation between *miR-451a* and *KIF2A* expression in LUSQ patients. TCGA database analyses showed that a negative correlation was detected between *miR-451a* and *KIF2A* expression in LUSQ patients (r = -0.180 and p = 0.010).

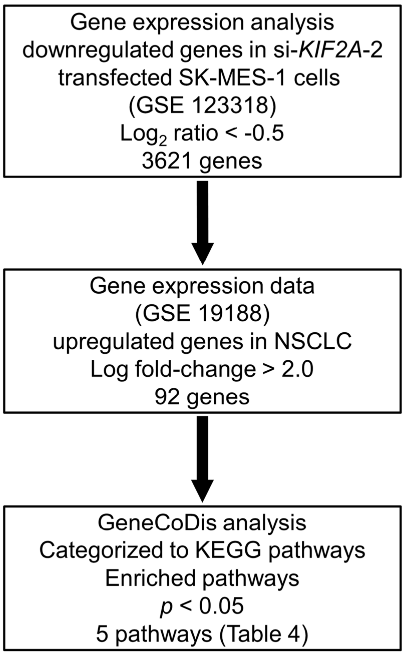

Figure S7. Flowchart illustration of the strategy for identification of *KIF2A*-mediated downstream pathways. By combination of genome-wide gene expression analyses and *in silico* analyses using si-*KIF2A*-transfected SK-MES-1 cells, we identified 5 pathways as significantly enriched pathways.

© 2019 by the authors. Submitted for possible open access publication under the terms and conditions of the Creative Commons Attribution (CC BY) license (http://creativecommons.org/licenses/by/4.0/).
